# Inferring Mental States via Linear and Non-Linear Body Movement Dynamics: A Pilot Study

**DOI:** 10.3390/s25226990

**Published:** 2025-11-15

**Authors:** Tad T. Brunyé, Kana Okano, James McIntyre, Madelyn K. Sandone, Lisa N. Townsend, Marissa Marko Lee, Marisa Smith, Gregory I. Hughes

**Affiliations:** 1 U.S. Army DEVCOM Soldier Center, Natick, MA 01760, USA; 2 Center for Applied Brain and Cognitive Sciences, Tufts University, Medford, MA 02155, USA; 3 Department of Electrical and Computer Engineering, Tufts University, Medford, MA 02155, USA; 4 U.S. Army DEVCOM Soldier Center Simulation and Training Technology Center (STTC), Orlando, FL 32826, USA

**Keywords:** inertial measurement units, optical motion capture, cognitive state estimation, workload, uncertainty, machine learning, movement dynamics

## Abstract

**Highlights:**

**What are the main findings?**

- This pilot study shows that body movement trajectories predict acute stress with moderate-to-high accuracy; workload and uncertainty were not reliably classified.
- The most informative movement-related features were primarily linear spectral and statistical measures, rather than non-linear chaos/complexity metrics.

**What is the implication of the main finding?**

- Whole-body movement sensing can serve as a low-burden indicator of acute stress for real-time sensing in the context of adaptive human–machine systems.
- Effective monitoring of workload and uncertainty may require larger training samples or multimodal fusion such as pairing with eye, voice, or peripheral physiology.

**Abstract:**

Stress, workload, and uncertainty characterize occupational tasks across sports, healthcare, military, and transportation domains. Emerging theory and empirical research suggest that coordinated whole-body movements may reflect these transient mental states. Wearable sensors and optical motion capture offer opportunities to quantify such movement dynamics and classify mental states that influence occupational performance and human–machine interaction. We tested this possibility in a small pilot study (*N* = 10) designed to test feasibility and identify preliminary movement features linked to mental states. Participants performed a perceptual decision-making task involving facial emotion recognition (i.e., deciding whether depicted faces were happy versus angry) with variable levels of stress (via a risk of electric shock), workload (via time pressure), and uncertainty (via visual degradation of task stimuli). The time series of movement trajectories was analyzed both holistically (full trajectory) and by phase: lowered (early), raising (middle), aiming (late), and face-to-face (sequential). For each epoch, up to 3844 linear and non-linear features were extracted across temporal, spectral, probability, divergence, and fractal domains. Features were entered into a repeated 10-fold cross-validation procedure using 80/20 train/test splits. Feature selection was conducted with the T-Rex Selector, and selected features were used to train a scikit-learn pipeline with a Robust Scaler and a Logistic Regression classifier. Models achieved mean ROC AUC scores as high as 0.76 for stress classification, with the highest sensitivity during the full movement trajectory and middle (raise) phases. Classification of workload and uncertainty states was less successful. These findings demonstrate the potential of movement-based sensing to infer stress states in applied settings and inform future human–machine interface development.

## 1. Introduction

Across occupational domains, ranging from sports to healthcare, military, and transportation, individuals operate in dynamic environments that frequently induce states of stress, variable workloads, and moments of uncertainty [1,2,3]. Stress often arises from threats or risks and can trigger heightened arousal, narrowed attentional focus, and impaired learning and memory [4,5,6]. Workload reflects the cognitive demands imposed by a task relative to an individual’s available resources, and excessive workload can impair performance (increasing errors and response latencies) by overwhelming attentional and executive systems [7,8,9]. Uncertainty occurs when information is ambiguous, incomplete, or conflicting, and it is associated with slower decision-making, reduced confidence, and strategic hesitation [10,11,12]. Due to these transient cognitive and affective states negatively influencing cognitive function and task execution [9,13,14,15,16,17], the accurate, real-time detection of these mental states can support adaptive systems that enhance safety, training, and performance under pressure. Such systems may include artificial intelligence (AI)-enhanced training simulators, stress- and workload-aware decision aids, or cognitive monitoring modules in occupational settings.

Traditional methods for cognitive state estimation, including sensors such as electroencephalography (EEG), functional near-infrared spectroscopy (fNIRS), and pupillometry, can offer valuable insights but often require instrumentation that can be cumbersome and sensitive to motion artifacts, limiting their applicability in naturalistic or mobile environments [18,19,20]. Increasingly, attention has turned to human movement sensing as a promising alternative for assessing cognitive states. Movement-based sensing offers a scalable and minimally intrusive alternative to conventional psychophysiological measures, enabling cognitive-state estimation in naturalistic, mobile settings. Rooted in embodied cognition, this approach assumes that mental states are not merely internal processes but are expressed through and shaped by the body. Behavioral cues, such as erratic gestures during confusion, postural stiffening under stress, or coordination shifts under cognitive load, reflect this link [21,22,23,24,25,26,27,28].

Sensor technologies, particularly optical motion capture (OMC) and inertial measurement units (IMUs), enable the high-resolution tracking of body or object movement. OMC systems use synchronized camera arrays to track markers in 3D space with sub-millimeter precision, ideal for simulated or constrained environments. IMUs offer mobile, lower-cost alternatives for field use, using accelerometers, gyroscopes, and magnetometers to track linear and angular motion. These sensing modalities enable the extraction of both linear features (such as velocity, acceleration, and positional displacement), and non-linear features that quantify signal complexity, predictability, and structure across time. Non-linear metrics such as entropy, fractal dimension, and Lyapunov exponents can capture chaotic or unstable movement patterns that may signal underlying changes in cognitive state. Together, linear and non-linear features offer a complementary view: linear metrics capture overt movement dynamics, while non-linear metrics may reveal subtle irregularities or fluctuations linked to mental processes. For example, recent research has demonstrated that non-linear features of walking, running, eye movements, brain activity, and heart rate have been useful for characterizing mental and physical states such as stress, workload, uncertainty, task competence, and postural stability [29,30,31,32,33,34,35,36,37,38]. Together, linear and non-linear features may provide unique value for mental state classification. Prioritizing these features is critical, affording input into machine learning pipelines that classify mental states with high temporal resolution and without the need for intrusive physiological instrumentation.

In occupational contexts, movement sensing can be used to quantify training progress, assess coordination, and analyze task performance [39,40]. However, few studies have examined whether dynamic whole-body movements, captured during task performance, can serve as a real-time proxy for evolving cognitive states such as stress, uncertainty, or workload [38]. Related work includes demonstrating postural immobility [41], posterior sway [42,43], or increased overall postural sway [44] when encountering aversive stimuli, and anterior sway when encountering positive or appealing stimuli [43,45]. Whereas this prior work focuses primarily on whole-body postural sway related to emotional experiences, herein we focus on the movement of occupational tools in response to three additional cognitive and affective states: stress, workload, and uncertainty. Identifying such movement-based indicators could enable early detection of cognitive strain, support adaptive training systems, and inform the development of human–machine interfaces for performance augmentation. These insights may also guide future integration of IMU-based sensing in wearable systems for occupational use.

The present pilot study addresses this gap by leveraging OMC to track movement trajectories while participants perform a decision-making task. Given the exploratory nature and small sample size (*N* = 10), the study was intended to establish feasibility and identify promising analytic approaches for future, larger-scale investigations. Participants experienced controlled manipulations of stress (via risk of electric shock), workload (via time pressure), and uncertainty (via stimulus clarity). Movement trajectories were segmented into task-relevant temporal phases, and several high-dimensional linear and non-linear features were extracted across temporal, spectral, probability, divergence, and fractal domains. Trajectory features were extracted using established Python libraries, and predictive models were evaluated using repeated cross-validation.

We had three main hypotheses. First, that stress would be most reliably classified from movement dynamics, relative to workload and uncertainty. Second, that predictive sensitivity would peak during the controller-raising phase, reflecting anticipatory arousal. And third, that both linear and non-linear features would contribute meaningfully to classification.

## 2. Materials and Methods

We conducted a human subjects experiment to test whether features quantified from movement dynamics afford classification of varied mental states. The study was approved by the institutional review boards at Tufts University and the U.S. Army Combat Capabilities, Army Research Laboratory (APPROVALS #24-089 and STUDY00005440).

### 2.1. Participants, Design & Procedure

A total of 18 (15 male, 3 female; Mean age 22.5) active duty military participants volunteered after providing written informed consent. Ten participants had complete datasets for analysis (8 male, 2 female; Mean age 23.3); data from eight participants were excluded due to incomplete motion capture records or missing behavioral data, resulting in 10 complete datasets suitable for analysis. Each participant completed two sessions on separate days, providing 20 sessions for analysis; during each session, they completed a perceptual decision-making task. Stress was manipulated in a within-participants manner: one session involved an acute stress induction, while the other did not, with the order counterbalanced across participants. During each session, participants experienced four conditions corresponding to the 2 × 2 within-participants design, with low versus high workload, and low versus high uncertainty conditions.

**Figure 1:**
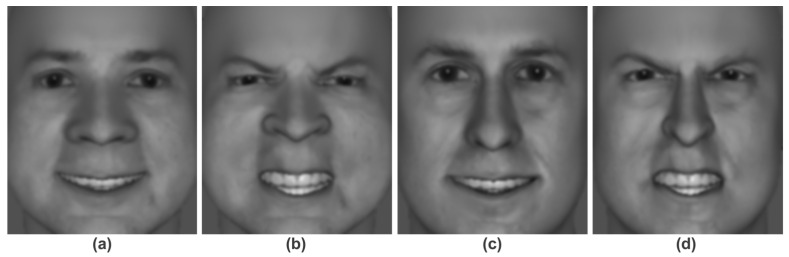
Two example faces with happy (panels (**a**,**c**)) versus angry (panels (**b**,**d**)) facial expressions.

In the stress session, participants wore a StressX Pro Belt (SETCAN Corporation, Winnipeg, MB, Canada) that randomly administered 4–8 electric torso shocks. Shocks were delivered using a current-limited stimulator (compliance up to 4.5 kV), with pulses of ≤150 ms, peak current limited to 1 mA, and mean delivered energy of 55 mJ per pulse. In the no-stress session, the belt instead delivered innocuous tactile vibrations. This level of torso shock is commonly used in research settings, is considered uncomfortable but not painful, and carries minimal risk to participant health and safety; however, it is sufficient to increase physiological stress responses [4,46,47]. During each session, participants performed four testing blocks of a task involving the discrimination of happy versus angry facial expressions (i.e., a perceptual decision-making task involving facial emotion recognition); a total of 200 white male faces were generated with the FaceGen Modeller software (v. 3.5; Singular Inversions, Inc., Toronto, ON, Canada). Note that we used only white male faces to limit potential influences of sex, race, and ethnicity on emotional decision-making; intensity of facial expressions was pilot tested to ensure reliable perceptual discrimination between the two classes (happy, angry), even under conditions of clarity masking. Example faces are provided in Figure 1. The four blocks corresponded to the 2 × 2 matrix of workload (low, high) and uncertainty (low, high) conditions. Condition order was randomized for each session.

Each block of the perceptual decision-making task included five minutes of trials, with each trial including a sequence of five faces. In a single trial, each of the five faces would appear sequentially from either left-to-right or right-to-left, on a large Cave Automatic Virtual Environment (CAVE) screen measuring 14′ wide by 8′ tall and running at 8K resolution (with the Unity engine [48]). Based on which of the four blocks the participant was experiencing, the faces appeared at different timing intervals corresponding to low (6 s) or high (3.6 s) workload, and at different visual clarities corresponding to low (50% clarity) or high (35% clarity) uncertainty. Timing intervals, clarities, and facial emotion intensities were determined via pilot study to ensure they similarly modulated accuracy across low versus high conditions. During trials, participants were instructed to classify each face as happy or angry and respond by aiming a controller (KWA PTS Centurion CM4 C4-10) at the face and either pressing a button (in response to a happy face) or pulling the trigger (in response to an angry face). Within a trial, faces needed to be responded to in order of appearance.

During each trial, participants would begin with the controller lowered, raise it in response to the first face appearing, and then move it laterally to aim at and respond to each face. The accuracy of each response was recorded alongside movements tracked with 6DOF (roll, pitch, yaw, and x, y, z position) at 20–25 Hz via OMC (TRACKPACK/E, Advanced Realtime Tracking, GmbH, Weilheim, Germany). Retroreflective markers were affixed along the longitudinal axis of the controller to capture translational and rotational motion, with two additional off-axis markers mounted at the end of the controller at distinct lateral offsets to disambiguate orientation. Trigger pulls were recorded by detecting a discrete pulse emitted from an IR laser integrated into the controller (Flash Kit distributed by Laser Ammo USA, Inc., Great Neck, NY, USA).

**Figure 2:**
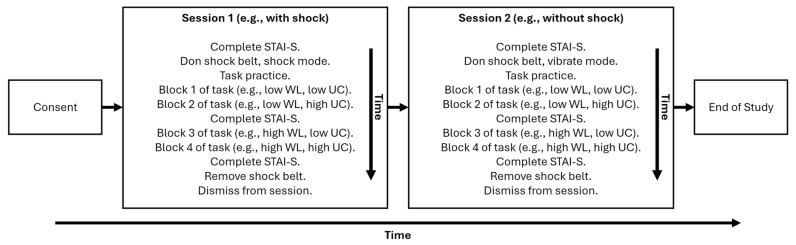
Summarized timeline of the overall experimental procedure, beginning with consenting and ending with dismissal. WL = workload, UC = uncertainty. Note that whether participants received shock or not was balanced across sessions, and block/condition pair order was randomized.

As manipulation checks, we measured three specific outcomes. First, we assessed the effectiveness of our stress induction by administering the state-trait anxiety inventory (STAI-S, [49]) before, during, and after task performance. Second, we evaluated task accuracy under the varied workload and uncertainty conditions (i.e., low, high) to determine if task performance was effectively degraded under high workload and uncertainty. The timeline of the overall procedure is depicted in Figure 2.

Additional research instruments were used but not analyzed or reported herein. These included eye-tracking glasses manufactured by SensoriMotoric Instruments (SMI; model SMI ETG2, Boston, MA, USA), a Zephyr Bioharness (Medtronic Healthcare, Boulder, CO, USA), the positive and negative affect schedule (PANAS, [50]), a custom fatigue scale, a virtual reality simulator sickness questionnaire (VRSQ, [51]), and salivary biosamples.

### 2.2. Data Processing

Data were collected from a total of 1649 trials. Each began with a lowered controller and proceeded with decisions being made for the sequence of five faces. Each trial’s time series of movement data was divided into multiple temporal epochs: an early lowered epoch, a middle raising epoch, a late aiming epoch, and face-to-face decision-making epochs (i.e., from face 1 to face 2, from face 2 to face 3, and so forth). This resulted in a total of 7773 epochs for analysis. From these, we removed 467 epochs (i.e., 6%) with data loss due to tracking error, and 63 trials (i.e., <1%) after manual visual inspection of trajectories for artifacts of body repositioning, scenario pauses, or other rare events. This resulted in a total of 7243 epochs for analysis.

**Figure 3:**
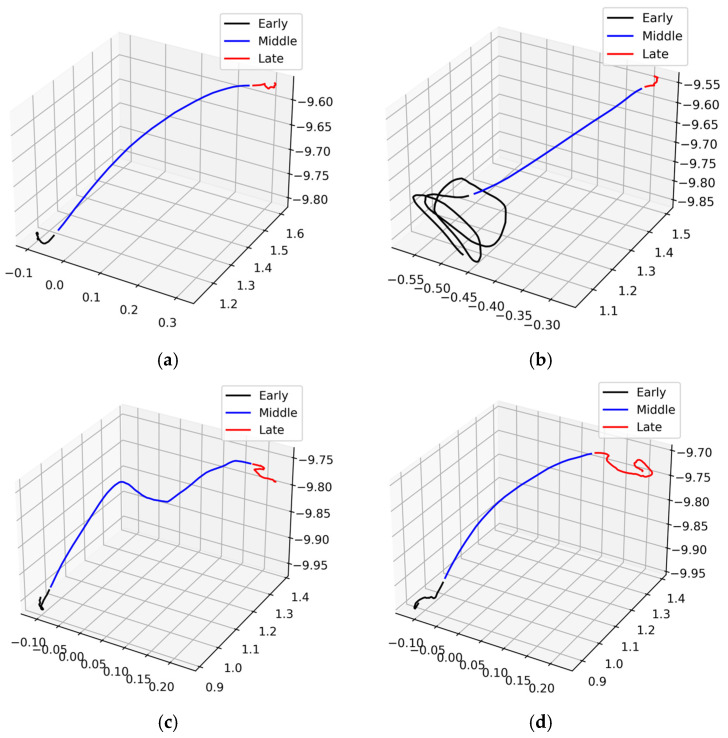
(**a**–**d**) Example time-series *raise* trial movement data, demonstrating the inherent variability of movement trajectories during *raise* trials, and parsed into early, middle, and late epochs.

To parse raise phases from the time series, we took a 6-s period before the first decision (i.e., button press or trigger pull). To parse the raise phase into early, middle, and late epochs, a dynamically set velocity threshold was applied per trial using a k-Means clustering algorithm (from scikit-learn) on the velocity magnitude with two clusters for movement and stillness. We then split each raise trial trajectory from the beginning of the first movement to the end of the final movement as the ‘raising’ period. This allowed us to capture the full raise segment, including any hesitations that may be present. Example raise trial movement trajectories are depicted in Figure 3.

**Figure 4:**
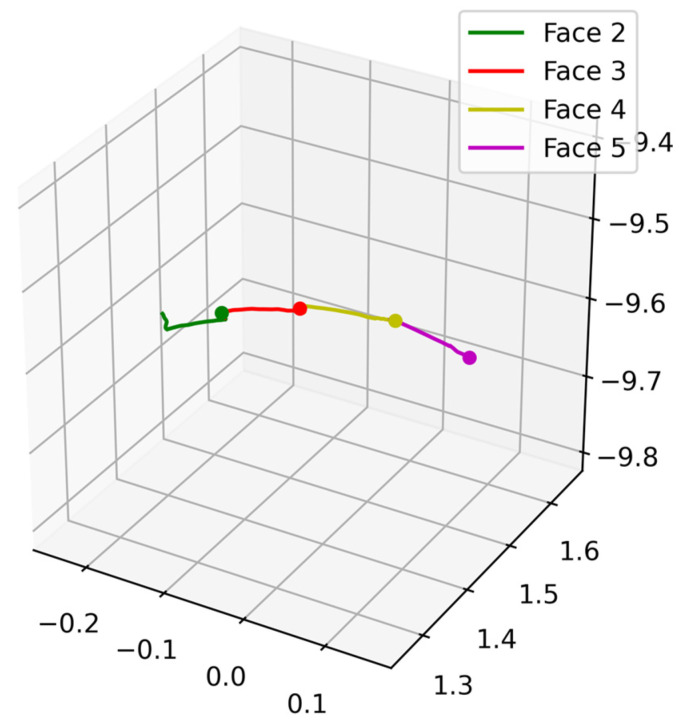
Example time-series *face-to-face* trial movement data for one consecutive set of faces.

To parse *face-to-face* epochs from the time series (i.e., Face 1 to Face 2, and so forth), we used the 3-s periods before each decision was made for faces 2–5 (note that the trajectory preceding Face 1 is included in the late aiming epoch). These inter-face epochs were collapsed into a single *face-to-face* epoch for analysis; if decisions were not made (e.g., trial timeout), these epochs were not used. Example face-to-face epoch movement trajectories are depicted in Figure 4.

These four *raise* and *face-to-face* epochs were carried forward for pre-processing movement trajectories. We did not analyze any trials where the participant did not respond to the stimulus given the lack of appreciable controller movement. Upon visual inspection of our data, all trials showed primarily translational rather than rotational movement (i.e., *x*-, *y*-, *z*-axes; predominately upward or lateral). Thus, analyses are restricted to the former.

To derive features from the 7243 epoch trajectories, we leveraged three Python (v3.12) packages: the Time Series Feature Extraction Library (TSFEL v0.2.0 [52]), the Time Series FeatuRe Extraction on basis of Scalable Hypothesis tests library (TSFRESH v0.21.1 [53]) and the Nonlinear Analysis Core’s NONANLibrary (v2.2.0.0 [54]). Together, these packages calculate features across the probability, temporal, divergence, spectral, and fractal domains. Parameters followed default implementations from each library’s documentation [52,53,54]. For example, TSFEL features were extracted with a 256-sample window and 50% overlap, and TSFRESH used standard hypothesis-testing parameters for relevance filtering. NONAN features were computed using established chaos and fractal estimators (e.g., Lyapunov exponent, approximate entropy). The corresponding GitHub pages of each package contain full feature lists. Features were calculated using velocity (V) and each of the X, Y, and Z translational axes, resulting in a maximum of 3844 features per temporal segment (i.e., early raise, middle raise, late raise, and face-to-face). For clarity, the Y axis reflects up/down (superior-inferior) movement, the X axis reflects left/right (medial-lateral) movement, and the Z axis reflects forward/backward (anterior–posterior) movement, all relative to the participant’s perspective.

Data analysis tested whether levels of stress (via threat of electric shock), workload (via time pressure), and/or uncertainty (via visual degradation) could be reliably classified from movement epoch features.

### 2.3. Data Analysis

Data analysis used a repeated (10 times) 10-fold cross-validation (i.e., 100 iterations), using 80% of the data for training and 20% for testing in each fold. We then used the Terminating Random Experiments Selector (T-Rex Selector) [55] for feature selection on the training set; this selector uses dummy variables to [38,56] control the false discovery rate (FDR), which was set at 0.05.

The features selected from the training set were used to construct a scikit-learn (version 1.4.0) pipeline consisting of a Robust Scaler followed by a Logistic Regression model. Using these selected features, predictions were generated with the trained pipeline. The logistic regression classifier used an L2 penalty with the ‘lbfgs’ solver, maximum 1000 iterations, and class-balanced weighting to account for any minor label imbalance. Parameters were selected based on conventional defaults in sci-kit-learn and validate in prior cognitive state classification studies using similar data scales [38,56]. To prevent data leakage across folds, all epochs from a given participant were assigned to a single partition within each iteration such that no participant contributed data to both training and testing sets, ensuring independence between training and test samples.

We selected logistic regression as our primary classifier due to its interpretability, stability in small-sample contexts, and reduced risk of overfitting relative to more complex algorithms. Given the modest size of our dataset, we did not compare multiple models, as doing so could yield inflated performance estimates from limited data. Future work with larger datasets will enable systematic benchmarking of alternative classifiers (e.g., SVM, random forest, or ensemble methods) to evaluate potential performance gains.

## 3. Results

Data from the three manipulation checks demonstrated generally successful stress and uncertainty manipulations. As a pilot study with a limited sample size, results should be interpreted cautiously and viewed as preliminary indicators of potential signal strength. The high stress condition increased STAI scores (M = 38.4, SD = 10.6) during the task, relative to the low stress condition (M = 33.2, SD = 7.72), with marginal significance, *F*(1, 9) = 3.13, *p* = 0.10. The uncertainty manipulation was effective, with high uncertainty conditions associated with significantly lower accuracy (M = 0.71, SD = 0.14) relative to low uncertainty conditions (M = 0.84, SD = 0.11), *F*(1, 9) = 63.19, *p* < 0.001. Finally, the workload manipulation led to numerically lower accuracy in the high (M = 0.76, SD = 0.14) versus low (M = 0.78, SD = 0.14) workload conditions, but this difference did not reach significance, *F*(1, 9) = 2.24, *p* < 0.17. A post hoc power analysis was conducted for the three manipulation check ANOVAs, is detailed in Table 1. Given the small sample size of this pilot study, the stress and workload manipulations were likely underpowered to detect medium effects, whereas the uncertainty manipulation achieved high power.

ROC AUC scores are provided in Table 2. For Stress, predictive models built on the full 6-s window of movement trajectory data achieved an average ROC AUC score of 0.76, indicating that trajectory information captured up to six seconds prior to the decision is effective for predicting stress levels. This predictive capability appears to be primarily driven by the lowered (early epoch) and raise (middle epoch) movements, which individually reached ROC AUC scores of 0.66 and 0.70, respectively.

**Figure 5:**
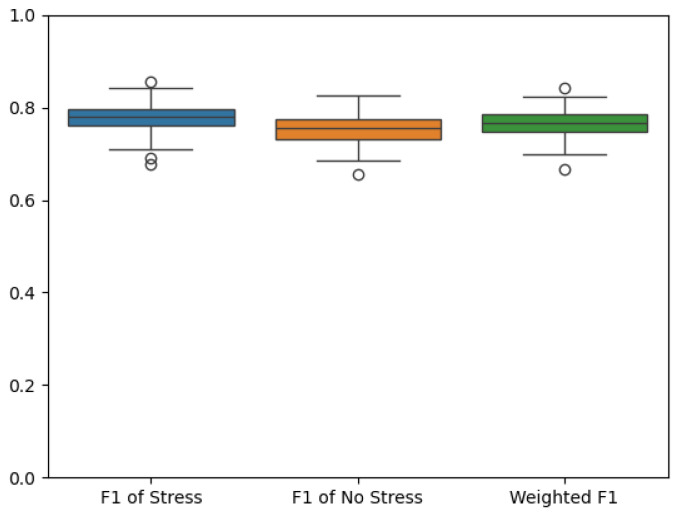
F1 scores from the 100 (10 repeated 10-fold) cross-validation iterations. Dots indicate outliers.

F1 scores from the 100 cross-validation iterations are depicted in Figure 5. The spread of scores indicates consistent model reliability, with limited outliers and a median that aligns with the overall mean. This stability suggests that the predictive model is generalizable across different subsamples of the data, rather than overfitting to any specific partition. Across iterations, classification metrics for stress averaged approximately 0.70–0.75 accuracy, 0.72 precision, and 0.70 recall, consistent with the observed ROC AUC of 0.76 and indicating overall balanced sensitivity and specificity across folds.

**Figure 6:**
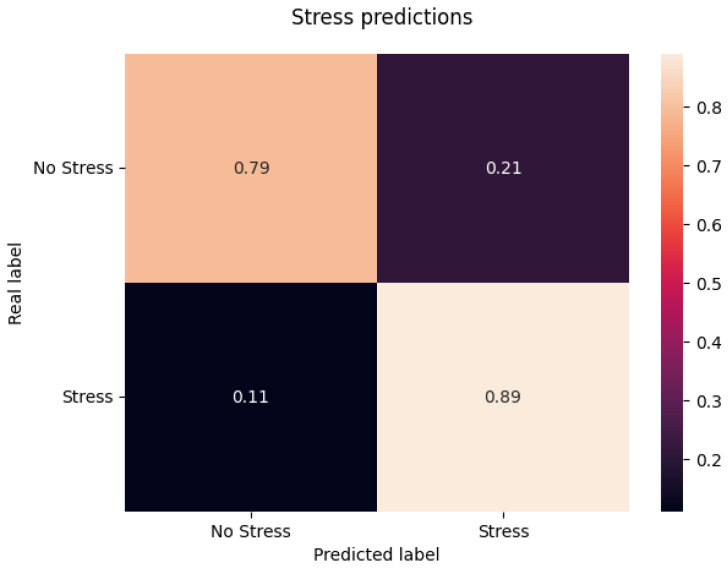
Confusion matrix from a sample cross-validation iteration, normalized over the true labels. The predictions from this iteration achieve an ROC AUC score of 0.84.

To illustrate model performance consistency, Figure 6 presents a representative confusion matrix from a cross-validation iteration, normalized over the true labels. This iteration achieved a notably strong ROC AUC score of 0.84, reflecting a particularly high discriminative capability. Of course, not all confusion matrices were this strong, as evidenced by the overall mean ROC AUC score of 0.76.

The most frequently selected features, from all iterations, are listed in Table 3; all the tabulated features were selected in 100% of iterations. Note that the most frequently selected features are derived from linear spectral analysis or basic statistical operations on the time series. While some involve non-linear mathematical transforms (e.g., mel scaling, absolute values), none are non-linear dynamical features in the sense of chaos or complexity metrics such as entropy or Lyapunov exponents.

A key feature emerging across all iterations is the Spectrogram mean coefficient at 7.26 Hz, extracted from the velocity magnitude signal using the TSFEL package. This feature consistently exhibited a positive coefficient in the Logistic Regression model, indicating that elevated values at this frequency are predictive of increased stress. Note that other consistently selected features (Table 3) showed variably positive or negative coefficients, making them less straightforward to explain.

**Figure 7:**
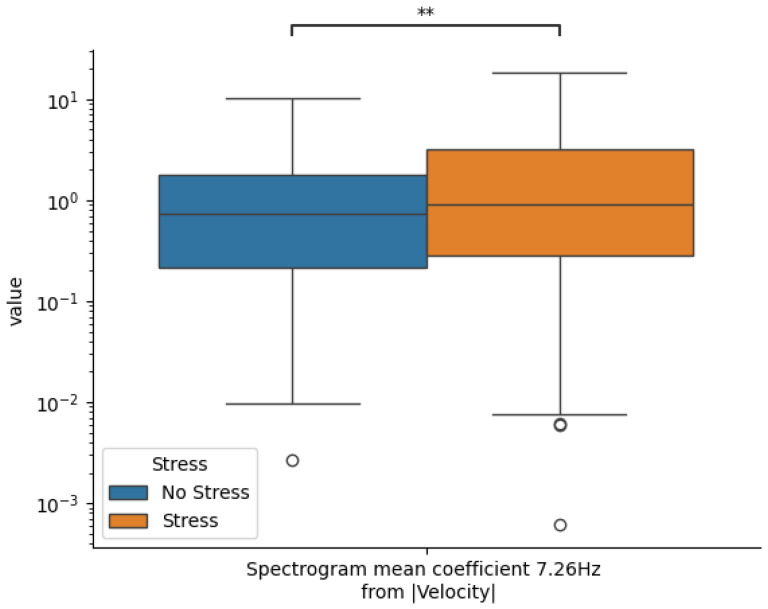
Box plot distinguishing stress and no-stress conditions for a notable feature. Including 95% confidence intervals and an indication of pairwise statistical significance (** *p* < 0.001). Dots indicate outliers.

Figure 7 presents a boxplot comparing the distributions of this key feature across stress and no-stress conditions. The figure includes 95% confidence intervals and highlights statistical significance at the *p* < 0.001 level. The clear separation between the two groups reinforces the feature’s utility as a discriminative marker of stress. Physiologically, this may correspond to subtle muscular tremors associated with posture maintenance, which are known to intensify under stress conditions. While speculative, this interpretation is supported by existing literature and aligns with prior work demonstrating the impacts of acute stress on motor control and tremors [57,58,59,60].

In contrast, models predicting Uncertainty and Workload did not yield significant performance. As shown in Table 2, ROC AUC scores for these conditions rarely exceeded 0.57, and in some cases, feature selection failed entirely, suggesting either a lack of distinguishable signal within the movement data or limitations in capturing these psychological states through trajectory-based features alone.

## 4. Discussion

Results of this initial pilot study demonstrate that high-resolution movement dynamics, captured via OMC, can serve as reliable indicators of acute stress during a simulated perceptual decision-making task involving facial emotion recognition. Given the modest pilot sample size (*N* = 10) and power analysis outcomes, these findings should be interpreted as preliminary and hypothesis-generating. Larger samples will be required to confirm the reliability of the observed stress-related movement patterns. Using a rich set of linear and non-linear features extracted from temporally segmented trajectories, we were able to classify stress states with moderate to high accuracy (ROC AUC = 0.76), particularly during the early and middle epochs. In contrast, classification models for uncertainty and workload failed to reach meaningful predictive performance; this was not particularly surprising given that these two variables were not robustly manipulated. That limitation aside, this pattern could suggest that movement-based indicators are more tightly coupled with stress-related arousal than with task-related ambiguity or cognitive demand in this context.

The finding that movement trajectories, particularly during the early and middle stages of the controller raise, were predictive of stress aligns with the embodied cognition framework, which posits that mental states are reflected in motor behavior [26,61,62]. The consistent emergence of a velocity-domain feature (Spectrogram mean coefficient at 7.26 Hz) as a reliable stress marker may point to subtle oscillatory dynamics or microtremors induced by heightened neuromuscular tension under stress. Such stress-related movement variability likely reflects increased sympathetic activation affecting fine motor stability, consistent with prior evidence linking arousal to motor jitter and co-contraction patterns [13,21,57,60]. The spectrogram mean coefficient reflects average signal power within a narrow frequency band, and elevated power near 7 Hz may indicate transient oscillatory instabilities or micromovements during posture transitions [58,60]. These frequency components may be linked to increased co-contraction of muscle groups or involuntary motor unit firing under stress, consistent with prior research showing that stress alters neuromuscular control strategies [63]. In the context of the raising phase, such perturbations could represent subtle tremor-like dynamics superimposed on otherwise smooth movement, offering a physiologically plausible marker of acute stress. These findings build upon prior work linking gross motor variability to stress and extend this literature by demonstrating sensitivity within a tightly controlled, goal-directed, upper-body movement context [21,22]. Notably, the stress signal was strongest during the raising phase, likely reflecting anticipatory arousal and preparatory control, rather than stimulus-driven decision-making [13,64]; it is also the longest continuous movement epoch, providing a rich time series that extended across vertical space. Early movement phases during perceptual decision-making tasks may be a window into pre-conscious motoric stress responses.

Contrary to our hypotheses, movement trajectories failed to yield reliable classifiers for workload and uncertainty. This likely reflects that the time-pressure manipulation, while effective in pilot testing, did not elicit a significant behavioral change in accuracy. Future studies should employ more immersive or sustained workload demands; for example, multitasking, dual-task tracking, or resource-sharing paradigms, which may evoke stronger workload effects, with downstream consequences for movement dynamics. This may reflect either limitations in how these mental states manifest behaviorally or inadequacies in the current sensing or feature extraction approach. While stress is known to elicit global changes in muscular tension, posture, and movement vigor, workload and uncertainty may influence more internal cognitive operations (such as attentional allocation, executive control, or decision latency) that are not readily detectable through pre-decisional gross motor features alone. Alternatively, the lack of predictive power could reflect the constrained movement space inherent in the controller-based task, which may have limited expression of compensatory strategies or behavioral adaptations to cognitive strain. It is also possible that workload and uncertainty modulate movement dynamics only under more prolonged, intense, or ecologically complex task settings, or that individual differences in working memory capacity or strategy use attenuated consistent expression of movement-based markers. Indeed, whereas our manipulation checks demonstrated the success of our stress and uncertainty manipulations, our workload manipulation numerically altered task accuracy but this difference was non-significant. More intense workload manipulations may lead to different results.

This study contributes to a growing body of work suggesting that whole-body or object-based movement trajectories can be used to infer internal cognitive-affective states [38,56,65,66,67]. Unlike traditional psychophysiological methods, movement tracking (particularly with IMUs) offers a scalable and minimally intrusive means of state assessment, particularly valuable in real-world occupational settings where other state sensing modalities may prove impractical. Our results highlight the utility of combining temporal segmentation with a broad feature extraction approach to capture both overt movement dynamics and hidden non-linear patterns. Importantly, our use of repeated cross-validation and a conservative feature selection approach (T-Rex Selector) reduces the risk of overfitting and provides confidence in the generalizability of findings across individuals and over time.

The specificity of movement-based features to stress, rather than workload or uncertainty, highlights the need to consider the phenomenology of each mental state and its likely behavioral correlates. Stress, as a physiological and psychological response to threat, appears more likely to manifest in muscle activation and subtle movement jitter than are states like uncertainty, which may involve more covert deliberative processes [10,17,38,60]. Future studies may benefit from multimodal approaches that pair movement data with eye tracking, voice analysis, or peripheral physiological signals to capture a broader array of cognitive-affective states [19,20,38].

Several limitations warrant caution in interpreting these findings. First, as a pilot study, the modest sample size and homogeneity of the participant pool may limit generalizability. The study was designed only to test analytic feasibility and identify candidate movement features for follow-on validation. Future work should increase participant numbers using high-throughput IMU-based data collection or multi-site collaborations to improve sample diversity (e.g., to balance male/female ratio, increase age range) and increase statistical power. Replication in larger, more diverse cohorts will be important for confirming robustness of our findings and population-level generalizability. Second, while the task was well-controlled, it may not reflect the full complexity of real-world contexts where stress, workload, and uncertainty co-occur dynamically and vary over longer timescales wherein neuromuscular fatigue may cooccur. Third, our labels for stress, workload, and uncertainty were derived from experimental manipulations rather than subjective or continuous self-reports, potentially limiting granularity and relevance to phenomenological expressions of these states. Integrating concurrent subjective or physiological measures would allow calibration between experimentally imposed and self-experienced stress, workload, and uncertainty. Fourth, although the predictive model performed well for stress, it remains correlational in nature, and the underlying mechanisms linking specific movement patterns to internal states require further elucidation, potentially via neurophysiological validation. Finally, our use of logistic regression prioritized model interpretability and stability over marginal accuracy gains from more complex algorithms. Future work with larger, more balanced datasets could explore ensemble and kernel-based methods to assess potential performance improvements.

Although the facial emotion recognition task provided precise experimental control, it represents a simplified abstraction of occupational performance. Translating these findings to dynamic, real-world settings will require adapting movement-sensing systems to unconstrained environments and integrating with context-aware data streams. Future work should explore how movement-related features interact with other sensor modalities, examine generalizability across tasks and populations, and evaluate the feasibility of real-time state detection in training settings and field-deployable systems using IMUs or camera-based systems. There is also promise in applying deep learning approaches capable of learning latent features from raw time series, although care must be taken to ensure interpretability and avoid overfitting. Indeed, the current analytic approach provides relatively high transparency and interpretability, identifying concrete linear and non-linear features that can be explored in future work.

## 5. Conclusions

In conclusion, this pilot study provides preliminary evidence that some cognitive states may be inferable from fine-grained features of movement during a stressful perceptual decision-making task. These findings support the potential of movement-based sensing as a low-burden method for real-time cognitive state monitoring and inspire future sensor integration into adaptive training or performance augmentation systems. The failure to classify workload and uncertainty points to the complexity of behavioral correlates of cognition and suggests the need for multimodal and task-specific sensing approaches. Overall, this work contributes to the development of next-generation human–machine systems that are sensitive to the fluctuating cognitive state of the user.

## Figures and Tables

**Table 1:** Results of post hoc power analyses conducted for the manipulation check ANOVAs (α = 0.05, *N* = 10), including partial eta-squared values and statistical power.

	F(1, 9)	η_p_^2^	f	Power
Stress	3.13	0.26	0.59	0.46
Uncertainty	63.19	0.88	2.71	0.99
Workload	2.24	0.20	0.05	0.35

**Table 2:** Mean ROC AUC scores for stress, uncertainty, and workload classification across movement epochs. AUC (area under the ROC curve) values reflect model discriminability, where 0.5 is chance and 1.0 is perfect classification. N/A indicates that the feature selector identified no discriminative features, which occurs when no voting level meets the requirement that the estimated false discovery proportion (FDP) remains below the target false discovery rate (FDR), suggesting either weak signals or true null conditions. * = this result is due only to task demands, which necessitates faster face-to-face responding in the high workload condition.

	Full Raise	Early Raise	Middle Raise	Late Raise	Face-to-Face
Stress	0.76	0.66	0.70	0.58	0.69
Uncertainty	0.57	0.55	N/A	0.53	0.56
Workload	0.54	0.50	N/A	0.53	0.94 *

**Table 3:** The most frequently selected features (selected in 100% of iterations), alongside a brief description of feature characteristics.

Feature	Analysis Package	Description
Spectrogram Mean Coefficient (7.26 Hz)	TSFEL	The mean spectral amplitude (at 7.26 Hz) for the velocity magnitude signal.
Spectral Amplitude Decrease	TSFEL	The average rate at which spectral amplitude declines from lower to higher frequencies.
Mel-Frequency Cepstral Coefficient	TSFEL	The 10th mel-frequency cepstral coefficient reflecting spectral envelope shape.
84th FFT Frequency (14 Hz) Magnitude	TSFRESH	Magnitude of the 84th discrete Fourier transform (FFT) coefficient (14 Hz) from the velocity magnitude signal.
Minimum of X-axis Value	TSFEL	Minimum observed value along the X-axis signal.
53rd FFT Frequency (8.83 Hz) Magnitude	TSFRESH	Magnitude of the 53rd discrete FFT coefficient (8.83 Hz) from the velocity magnitude signal.

## Data Availability

Given the highly specialized and potentially sensitive nature of participants (i.e., military personnel) and the relatively small sample size that can lead to unintentional identity ascertainment, deidentified data are only available by request to the corresponding author.

## Abbreviations

The following abbreviations are used in this manuscript:

AI	Artificial Intelligence
CAVE	Cave Automatic Virtual Environment
EEG	Electroencephalography
DEVCOM	Combat Capabilities Development Command
fNIRS	Functional Near-Infrared Spectroscopy
FDP	False Discovery Proportion
FDR	False Discovery Rate
FFT	Fast Fourier Transform
IMU	Inertial Measurement Unit
IR	Infrared
NONAN	Nonlinear Analysis Core
OMC	Optical Motion Capture
PANAS	Positive and Negative Affect Schedule
ROC	Receiver Operating Characteristic
AUC	Area Under the Curve
SMI	SensoMotoric Instruments
STAI	State-Trait Anxiety Inventory
TSFEL	Time Series Feature Extraction Library
TSFRESH	Time Series Feature Extraction on basis of Scalable Hypothesis tests
VRSQ	Virtual Reality Sickness Questionnaire
6DOFs	Six Degrees of Freedom
